# A robust superhydrophobic TiO_2_ NPs coated cellulose sponge for highly efficient oil-water separation

**DOI:** 10.1038/s41598-017-09912-9

**Published:** 2017-08-25

**Authors:** Hui Zhang, Yuqi Li, Zexiang Lu, Lihui Chen, Liulian Huang, Mizi Fan

**Affiliations:** 1 College of Materials Engineering, Fujian Agriculture and Forestry University, Fuzhou, 350002 China; 2 Nanocellulose and Biocomposites Research Centre, College of Engineering, Design and Physical Sciences, Brunel University, UB8 3PH Uxbridge, UK

## Abstract

Oil-water separation has recently become a worldwide concern because of the increasing oil spill accidents and industrial oily wastewater generation. Herein, a facile method with the combined superhydrophobic coating and adhesive was used to fabricate superhydrophobic TiO_2_ NPs coated cellulose sponge. The developed materials exhibited excellent superhydrophobicity (WCA = 171°) and superoleophilicity (OCA = 0°), which can separate a variety of oil-water mixtures, including chloroform, toluene, kerosene and other contaminations. A high separation efficiency up to 98.5% for chloroform-water mixture was achieved when used for gravity-driven oil/water separation test. More importantly, the as-prepared samples exhibited excellent chemical stability and mechanical abrasion resistance even towards various corrosive oil/water mixtures (such as strong acid, alkali solution and salt-water environment) or a strong abrasion by aluminium oxide sandpaper of 600 mesh. In addition, the separation efficiency remained above 93% even after 40 scratch cycles, and the materials could be reused with a stable hydrophobicity, indicating a strong potential for industrial application.

## Introduction

In the past decades, marine oil spillage and chemical leakage have caused destructive impact on the water environment^1–3^. Selective absorption^4^ and direct separation of oil/water mixture^5–7^ are two main processing methods to deal with water pollution issues. Among those efficient and effective methods^8–10^ reported, the direct separation has been considered the optimal. For example, the oil/water mixture could be separated into two distinguished phases after a period of static storage because of the different densities of water and oil. However, the development of cost-effective, highly efficient and scalable separation materials, e.g. separation efficiency >98%, to achieve a rapid oil/water separation is desperately needed.

Constructing a super-antiwetting surface^11–14^ with superhydrophobicity and superoleophilicity has recently drawn great attentions. Oil droplets could be quickly absorbed and permeated through this kind of surface, while water droplets were repelled completely. Therefore, this super-antiwetting surface has potential applications in the field of oil/water separation. Jiang *et al* ^15^. firstly fabricated a mesh film by a spray coating to separate the oil-water mixtures. Due to its special wettability, the mesh film exhibited excellent oil/water separation efficiency and selectivity. By spraying the mixture of palygorskite and polyurethane on copper mesh, Li *et al* ^16^. also fabricated an underwater superoleophobic mesh film, which has high oil/water separation efficiency (up to 99%). With these inspirations, a variety of techniques^17–21^ have been developed to produce oil/water separation materials with super-antiwetting surfaces, including bio-based foam membranes^22^, filter paper^23^, metallic mesh-based materials^24–26^ and ceramic microfiltration membranes^27^, *et al*. Although these materials exhibited excellent oil-water selectivity and separation efficiency, there remained a number of problems, including the complicated preparing methods, expensive raw materials, poor mechanical stability and chemical stability, which limited their large-scale production.

Currently, cellulose-based oil/water separation materials have attracted wide attention due to its low cost, biodegradability and renewability. The super-antiwetting surface could be realized by constructing a highly textured structure on cellulose surface and introducing a low surface energy substrate^28^. Various fabrication methods have been reported to construct this kind of surfaces, including the plasma treatment method^29^, sol-gel method^30, 31^, chemical vapor deposition^32^, layer-by-layer technique^33^, Pickering emulsion polymerization^34^, spray-coating method^30^, and so forth. Among them, the spray-coating is considered commercially available for large-scale industrial application and independent of the substrate characteristics such as shape, surface structure and electrical conductivity^35^. However, the coatings are traditionally sprayed directly onto the material surface, which will easily cause the falling-off of the micro/nanostructures in application. The as-prepared antiwetting surface is inherently fragile and could not be used towards a strong mechanical friction and abrasion, although some nanoparticles (NPs) of coating may be embedded into the rough fabric surface. Furthermore, most of reported fabric materials for oil/water separation could not be used towards a harsh environment (such as strong acid, alkali solution and salt-water environment).

This research fabricated a superhydrophobic TiO_2_ NPs coated cellulose sponge via a facile method with the combined superhydrophobic coating and adhesive. This “superhydrophobic coating + adhesive” method was, to our knowledge, used for gravity-driven oil-water separation for the first time. The performance properties of the as-prepared material, including wettability, chemical stability, mechanical abrasion resistance, separation efficiency for oil-water mixtures and reusability were investigated in detail. As expected, the developed materials have a super-antiwetting surface, which can selectively capture the oil successfully while repelling the water completely. When used for gravity-driven oil-water separation test, the materials showed a high separation efficiency. More interestingly, the developed material exhibited excellent chemical stability, mechanical abrasion resistance and reusability.

## Results and Discussion

**Figure 1:**
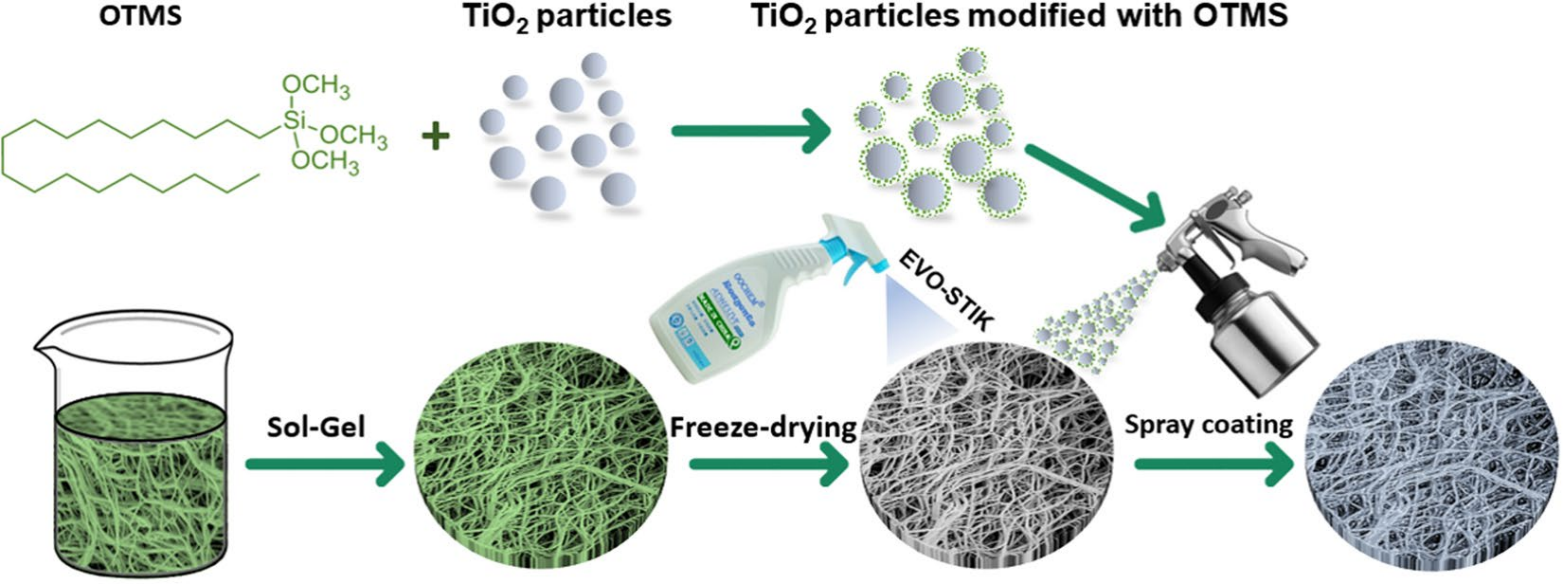
Schematic illustration of the superhydrophobic cellulose sponge production using a spraying method.

The superhydrophobic TiO_2_ NP_S_ coated cellulose sponge fabricated by a facile “superhydrophobic coating + adhesive” method was shown in Fig. 1. Two kinds of TiO_2_ nanoparticles with different sizes (~100 nm and ~25 nm) were mixed to construct the hierarchical rough structure on the cellulose sponge. Meanwhile, the adhesive (EVO-STIK) was sprayed to increase the binding force between the superhydrophobic TiO_2_ NP_S_ and cellulose sponge.

**Figure 2:**
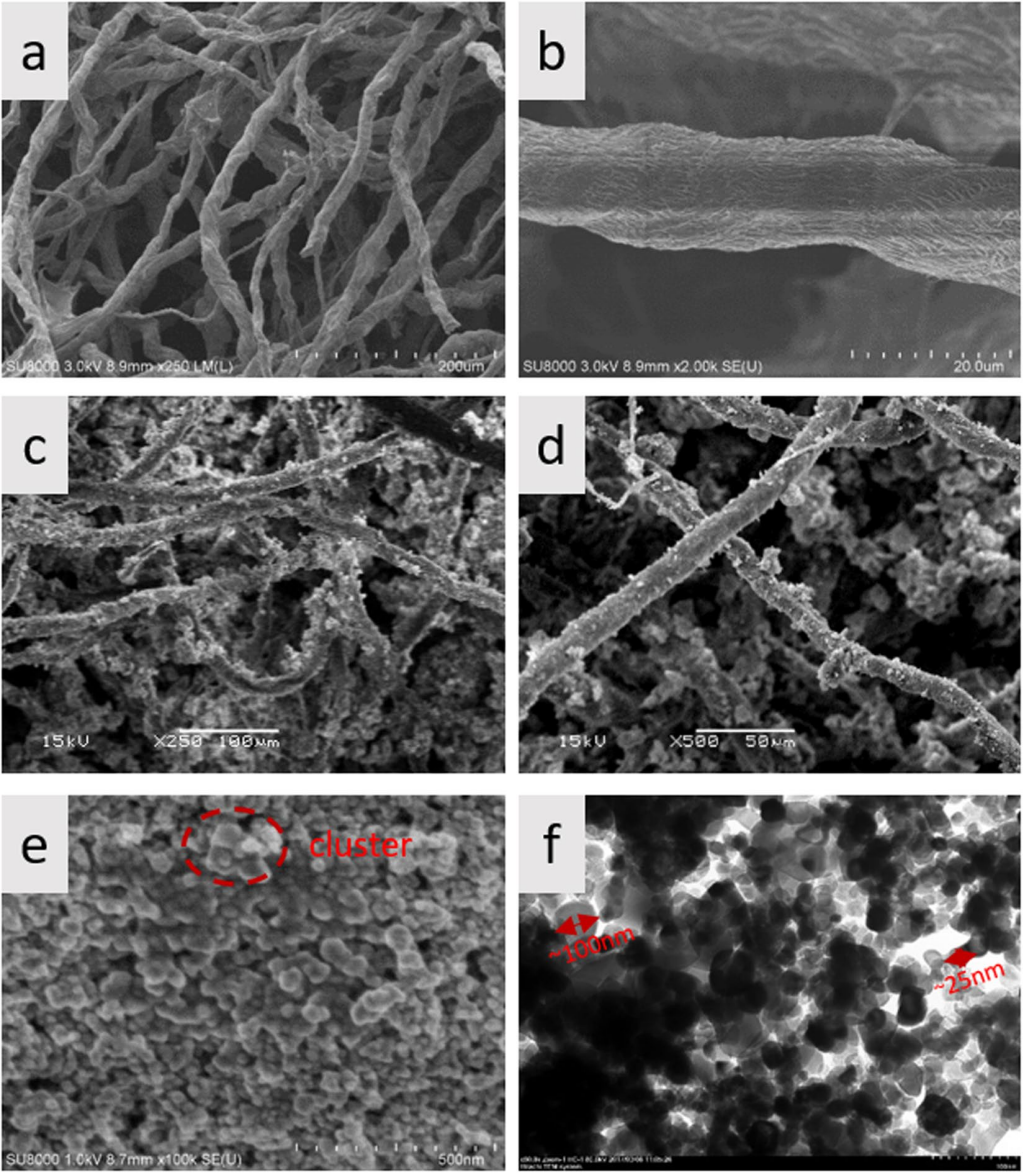
SEM images of (**a**) the original cellulose sponge, (**b**) single fiber, (**c**) TiO_2_ NPs coated cellulose sponge, (**d**) TiO_2_ NPs coated single fiber, (**e**) the as-prepared TiO_2_ superhydrophobic coating, and (**f**) TEM image of TiO_2_ superhydrophobic coating.

The surface morphologies of the original and the TiO_2_ NP_S_ coated cellulose sponge were analyzed by FE-SEM, and shown in Fig. 2. As can be seen from Fig. 2a, the as-prepared cellulose sponge has an open porous network with uniform fiber of 20 um in diameter (Fig. 2b), and the pore size of network is 20~100 um. As shown in Fig. 2c and d, the treated fibers are coated with TiO_2_ NPs. The low-magnification image in Fig. 2c showed that the TiO_2_ NPs are randomly distributed and close-packed over all treated fabrics and aggregated around the spaces of interfibers of sponge. High-magnification FE-SEM image in Fig. 2e showed that some TiO_2_ NPs modified with OTMS aggregated into micro/nano-cluster, resulting in the hierarchical roughness of micro/nano structure on the coating and around the spaces of inter-fibers (Fig. 2c). This aggregation might be due to the grafting of long chain alkyl group on the surface of TiO_2_ NPs. It is evident that this roughness of micro/nano structure is essential for the superhydrophobicity of the resulted materials. TEM image in Fig. 2f clearly showed the constituent nanoparticles in the coating. It is apparent that two kinds of TiO_2_ nanoparticles of different sizes randomly distributed in the superhydrophobic coating, forming the special rough structure. The size of the TiO_2_ nanoparticles was about 25 nm and 100 nm, respectively.

**Figure 3:**
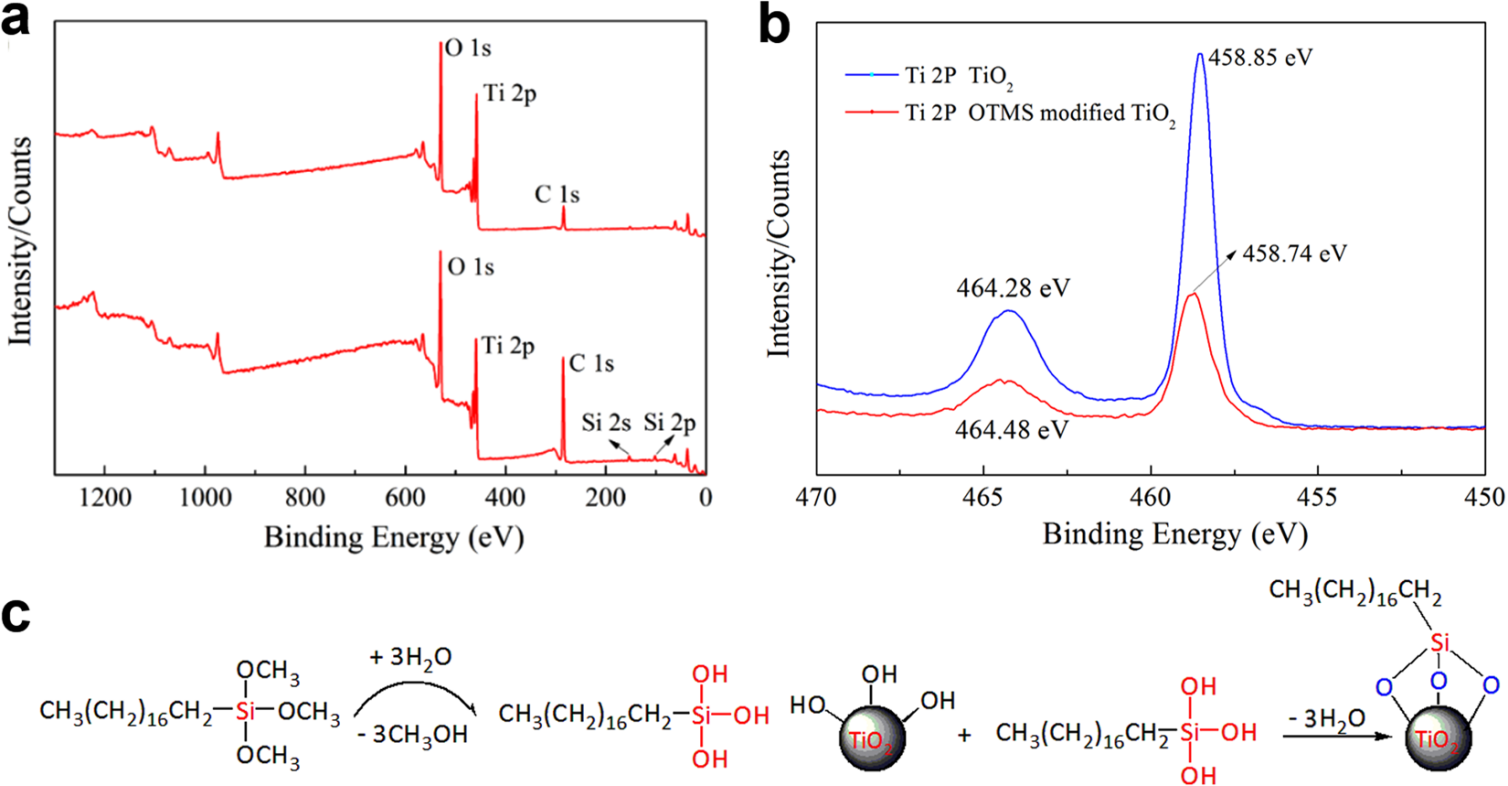
(**a**) The XPS spectra of (I) the original and (II) OTMS modified TiO_2_ NPs, (**b**) The XPS narrow scan for Ti 2p, (**c**) A schematic illustration of the OTMS modification mechanism in the silanization reaction.

The chemical compositions of the original and superhydrophobic TiO_2_ NP_S_ coated cellulose sponge were determined by X-ray photoelectron spectroscopy (XPS). It can be seen that the spectrum of the original TiO_2_ nanoparticles (I) showed peaks for oxygen, titanium and carbon (Fig. 3a). However, besides C1s peaks, the Si 2p and Si 2 s peaks appeared in the spectrum of OTMS modified TiO_2_ nanoparticles (II). Meanwhile, the relative intensity of C1s peaks has enhanced significantly compared with the spectrum of the original TiO_2_ nanoparticles. It also can be seen from Fig. 3b that the chemical environment of Ti has changed after OTMS modification, with the binding energy of Ti 2p varied from 458.85ev to 458.74ev, indicating that the TiO_2_ NP_S_ has been successfully modified by OTMS. In addition, the OTMS modification process may be proposed as shown in Fig. 3c. Firstly, –Si–OH functional groups were produced by the hydrolysis of OTMS. Then, the chemical reaction occurred between the –Si–OH and the hydrophilic –OH functional groups in TiO_2_ NP_S_. The –OH functional groups in the TiO_2_ NP_S_ were replaced by long chain alkyl group in OTMS, resulting in the hydrophobicity of TiO_2_ NP_S_.

The CA values were measured to identify the hydrophobicity and oleophilicity of superhydrophobic TiO_2_ NP_S_ coated cellulose sponge. It can be seen from Supplementary Fig. S1a that the water and oil droplets could penetrate into the uncoated cellulose sponge, due to a great number of hydroxyl groups in cellulose fibers. After coated with the superhydrophobic TiO_2_ NP_S_, water droplets could stand on the cellulose sponge surface (Supplementary Fig. S1b) forming a highly spherical bead (WCA = 171°), while the oil droplets were absorbed quickly (OCA = 0°), indicating excellent superhydrophobicity and the superoleophilicity of the as-prepared cellulose sponge. In order to better understand the dynamic wettability of the fabricated cellulose sponge, the adhesion and permeating process of water and oil droplets were recorded by a high-speed camera system (Supplementary Fig. S2). Figure S2a showed the pictures of water droplet (5 μL) touching and then leaving the sample surface. As can be seen from Fig. S2a, the water droplet was forced to contact the sample surface with an obvious deformation, and almost no deformation was seen when leaving the sample surface, indicating an extremely low adhesion for the water droplets. This low adhesion is very favorable for oil-water separation process. It also can be seen from Supplementary Movie S1 and S2, after being dropped onto the fabricated cellulose sponge, the water droplets slipped quickly and could not stick on the sample surface. It was considered to be in the Cassie state, and this phenomenon was similar to self-cleaning lotus leaf, which could be explained by the rough micro/nano structure and low surface energy substrate on the fabricated surface of cellulose sponge. Simultaneously, the oil adsorption process was shown in Fig. S2b. When a 5 μL oil droplet (pump oil) contacted the cellulose sponge surface, it spread out quickly and penetrated into the sample within 1.2 s, indicating an excellent oil wetting of the surface.

The oil-water selectivity of the as-prepared cellulose sponge was also investigated in this study. Figures S3a and S3b showed the absorption processes of the as-prepared sample for light oil (soybean oil) on the water surface and heavy oil (chloroform) underwater, respectively. After being immersed into oil/water mixture, the sample strongly repelled the water but absorbed the soybean oil and chloroform (dyed with yellow) selectively and instantaneously as soon as it touched, indicating that the sample has excellent oil selectivity and adsorption capacity.

**Figure 4:**
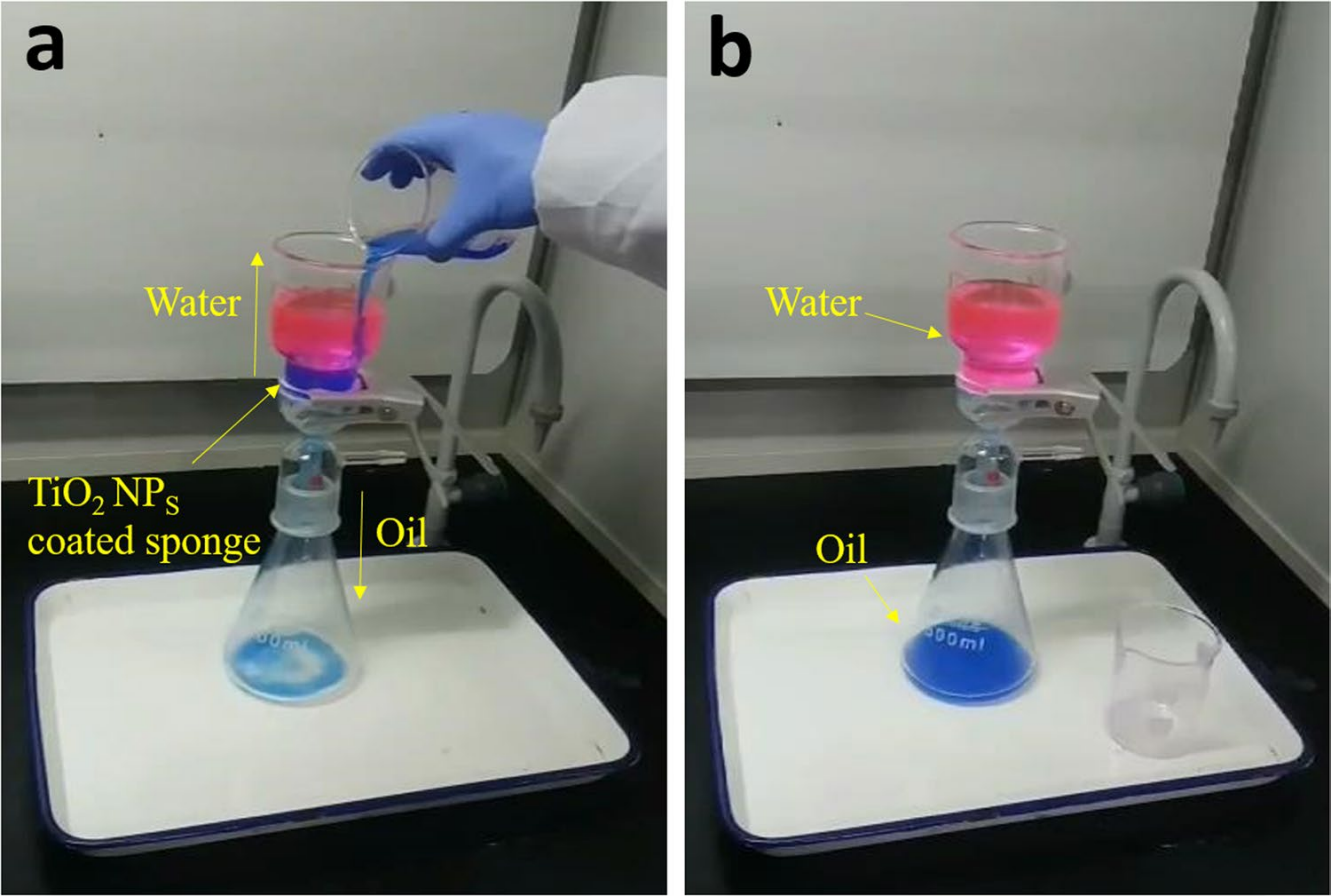
Oil/water separation process of the developed cellulose sponge (oil dyed with blue for enhancing the visual effect): (**a**) before separation, (**b**) after separation.

The oil-water separation experiment was performed by taking chloroform-water mixture as an example. As can be observed from Fig. 4, driven by its own gravity, the oil dyed with blue (chloroform) penetrated through the pre-wetted superhydrophobic cellulose sponge quickly, while the water dyed with red was selectively blocked (Supplementary Movie S3).

The reasons for this phenomenon may be explained as follows: when the superhydrophobic cellulose sponge was pre-wetted by oils or organic solvent, the hierarchical rough structure of the surface was filled with oil, as a result, the sponge surface turned relatively flat. In this case, the water droplets tended to be in the Wenzel state with a high contact angle hysteresis after being dropped onto the wetted surface^36^. Meanwhile, the low resistance of the wetted surface also causes the water droplets to move away easily. After separation, no visible water was observed in the filtered oil, indicating a high oil-water separation efficiency. According to the Equation *φ*(%) = *m*_1_/*m*_0_ × 100 (where, *m*_0_ and *m*_1_ are the mass of the water before and after the separation process, respectively), the calculated separation efficiency for chloroform-water mixture is over 98.5% and 92.0% for other oils and organic solvents respectively (Supplementary Fig. S4).

**Figure 5:**
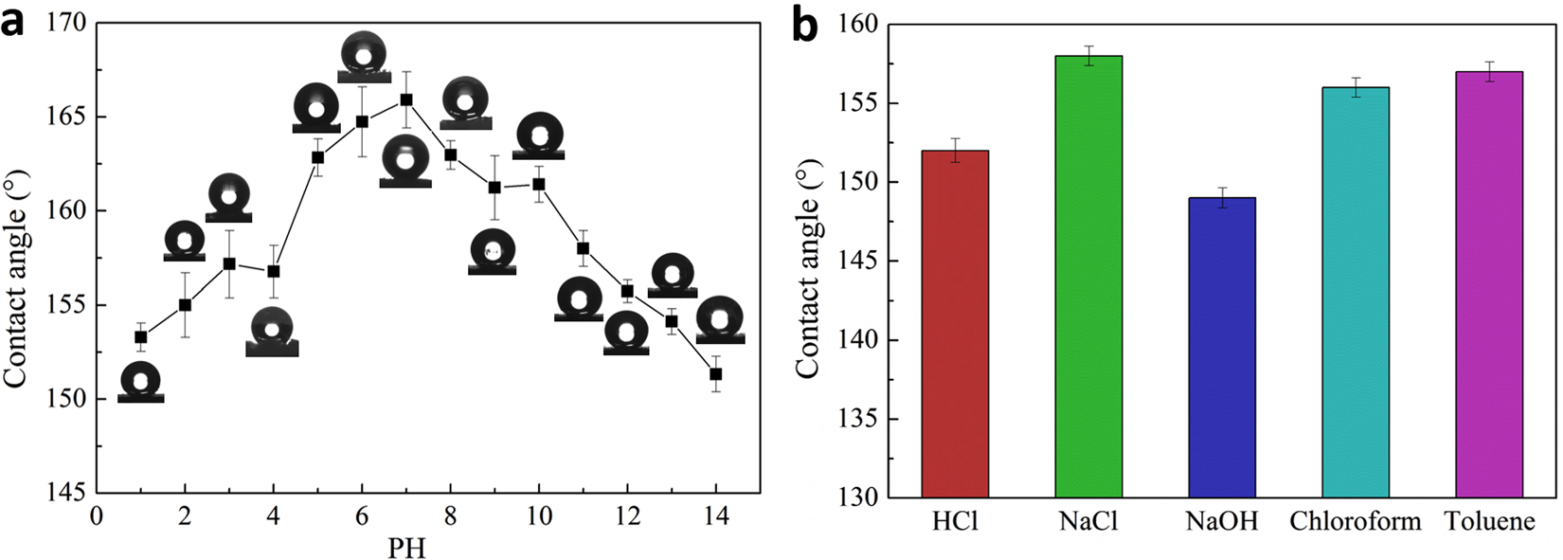
Relationship between the water contact angle with (**a**) pH values and (**b**) corrosive medium liquids, salt solution and organic solvent.

The variation of water contact angle with the PH values was shown in Fig. 5a. Within the experimental error, the water contact angles were all greater than 150° in all PH values ranging from 1 to 14, indicating that the as-prepared cellulose sponge had excellent superhydrophobicity. When the samples were immersed into the corrosive solution (1 mol/L HCl and 1 mol/L NaOH), organic solvent (chloroform and toluene) and 1 mol/L NaCl for 24 h respectively, they were still showed stable hydrophobicity. As shown in Fig. 5b, most of the water contact angles were higher than 150°, except that the water contact angle was 149° in 1 mol/L NaOH solution. All the above test results showed that the as-prepared superhydrophobic sponge has a robust chemical stability. It not only has a wide range of PH feasibility, but also exhibits excellent resistance to many corrosive liquids like strong acid, alkali, salt solution and organic solvent, which are significantly important for industrialization of the as-prepared sample.

**Figure 6:**
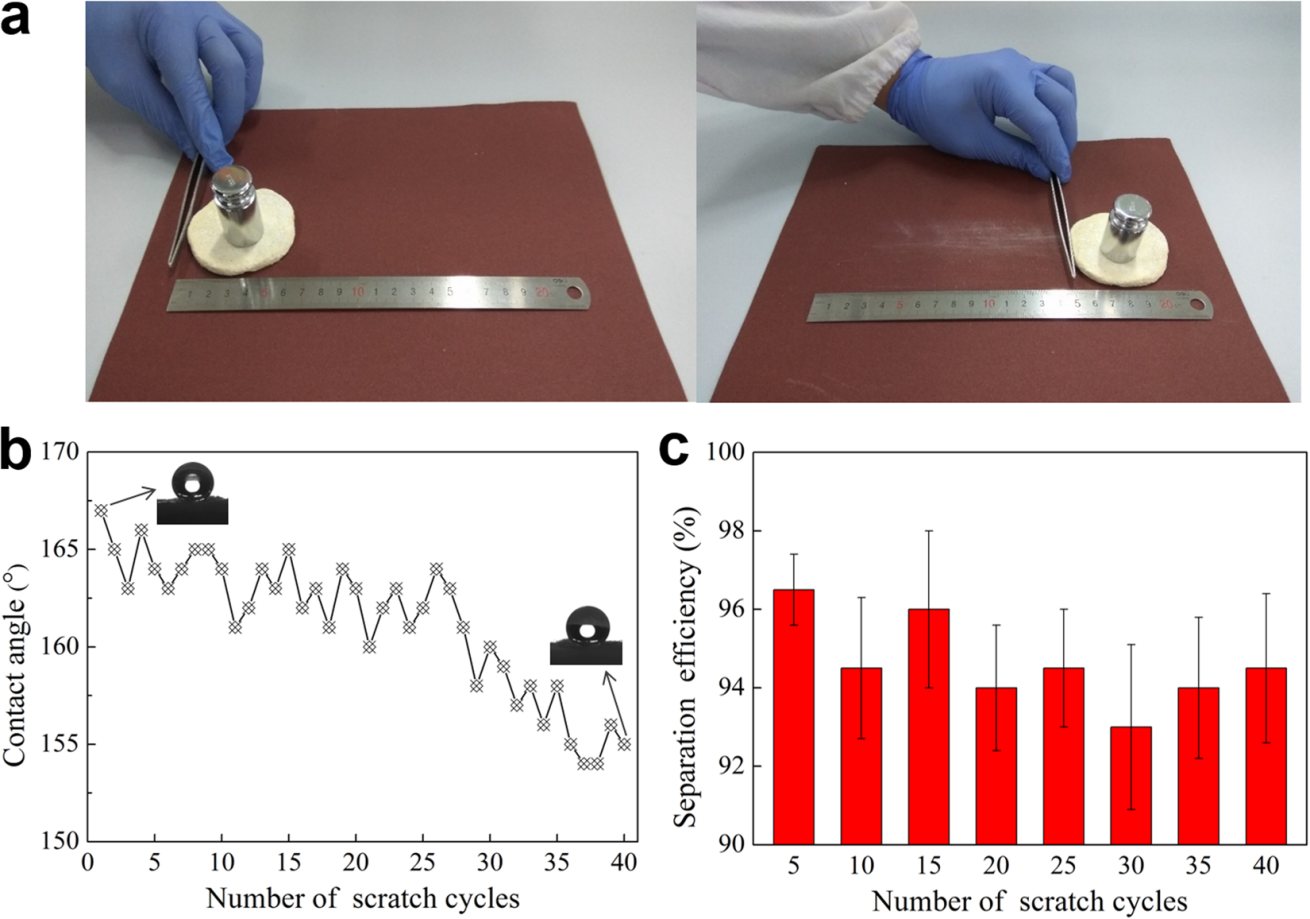
(**a**) Sandpaper abrasion test of the developed superhydrophobic cellulose sponge (one cycle of the test), (**b**) plot of water contact angles and the number of scratch cycles, (**c**) separation efficiency versus number of scratch cycles (chloroform-water mixture as a test).

Scratch test was considered to be an effective method to evaluate the robustness of the as-prepared super-antiwetting surface against the mechanical force^37–39^. The aluminium oxide sandpaper of 600 mesh was used as an abrasion surface during the scratch test. As shown in Fig. 6a, under a weight of 100 g, the developed sample was abraded by sandpaper for 40 cycles. In the sandpaper abrasion test, the TiO_2_ NPs coated cellulose sponge was slightly damaged, a few nanoparticles and tiny fibers were abraded out (Supplementary Movie S4 and Fig. S5b). As shown in Fig. 6b, the water contact angle values decreased with the increase of the number of scratches cycles, but they were still higher than 150° after 40 scratch cycles, indicating excellent mechanical abrasion resistance of the as-prepared super-antiwetting surface. Fig. 6c displays the changes of separation efficiencies as a function of scratch cycles during the abrasion test. The result showed that the separation efficiency of chloroform-water mixture still remained above 93% after 40 scratch cycles. The above test demonstrated that the as-prepared sample has excellent robustness. This robustness may be ascribed to the adhesive and coarse structure of cellulose sponge, which may make the nanoparticles fixed and embedded into the fibers firmly (see Supplementary Fig. S5 and Fig. S6). As a result, most of the TiO_2_ NPs left on the sample surface and the spaces of inter-fibers of sponge, although a few have fallen off in the process of sandpaper abrasion.

The recyclability of the as-prepared superhydrophobic cellulose sponge was also evaluated in this study. After being rinsed with ethanol and water thoroughly, the cleaned cellulose sponges were dried for the next use. Fig. S7 showed the changes of water contact angle with the recycle numbers of the superhydrophobic cellulose sponge. It can be seen from Fig. S7 that, with the increase of the recycle numbers from 1 to 25, the CA values decreased gradually, which might be caused by the fall off of a small amount of TiO_2_ superhydrophobic coating. However, the CA values were still nearly 150°, indicating that the recycled samples remained the stable hydrophobicity and were feasible for the large-scale industrial applications.

In conclusion, a robust superhydrophobic TiO_2_ NPs coated cellulose sponge was fabricated via a facile “superhydrophobic coating + adhesive” method. This simple method has successfully overcome the loss off nanoparticles in their subsequent uses. The developed sample exhibited excellent super-antiwetting property (WCA = 171°and OCA = 0°) and was feasible for gravity-driven oil/water separation uses, such as chloroform, toluene and kerosene. A high separation efficiency up to 98.5% for chloroform-water mixture was achieved in the test and a good reusability could be seen. More interestingly, the sample exhibited excellent chemical stability and mechanical abrasion resistance even towards various corrosive oil/water mixtures or a strong abrasion. The developed approach is feasible for mass industrial production.

## Materials and Methods

### Materials

Titanium oxide nanoparticles (~100 nm in diameter) and TiO_2_ P25 were purchased from Aladdin Biochemical Technology Co., Ltd. (Shanghai, China). Octadecyltrimethoxysilane (OTMS) was obtained from Macklin Biochemical Co., Ltd. (Shanghai, China). Absorbent cotton was obtained from a local pharmacy and ball-milled before use. EVO-STIK was obtained from Bostik Co. (British, Europe). Kerosene and Soybean oil were supplied by Zhaoming Trading Co, Ltd (Fuzhou, China). Toluene, Chloroform, and Hexane were purchased from Sinopharm Chemical Reagent Co., Ltd. (Shanghai, China, purity 99%). All chemicals were analytical grade reagents and were used without any further purification.

### The preparation methods

#### Preparation of superhydrophobic coating

In brief, 1.58 ml of OTMS was placed into 201.6 ml of absolute ethanol, to which 8.4 g of P25 (TiO_2_ NP_S_, ~25 nm) was added and magnetically stirred for 2 h. Then, 8.4 g of titanium oxide nanoparticles (TiO_2_ NP_S_, ~100 nm) were added to the above mixture and stirring continued for 0.5 h. Finally, the above mixture was kept under ultrasonic irradiation with a power of 150 w for 2.5 h until a uniform paint-like suspension was formed.

#### Preparation of cellulose sponge

Sodium hydroxide/urea solution system (7 wt%/12 wt%) was prepared and placed in a low temperature water tank for freezing until −12 °C was achieved. Thereafter, the absorbent cotton of 1.46 g was weighed and added to the above solution system slowly with the mechanical stirring for 2 h. The resulting homogeneous solution was poured into the cylindrical polystyrene mould. After regeneration in deionized water, the samples were placed into the freeze-drying system (Telstar LyoBeta, Spain) for drying 6 h to produce the cellulose sponge.

#### Preparation of superhydrophobic cellulose sponge

Subsequently, by using a high-pressure spray gun (with 0.2 Mpa N_2_), the as-prepared paint-like suspension was sprayed onto the sponge surface, which has been treated by the spray adhesive (EVO-STIK) previously. The spraying process was repeated 10~15 times. Finally, the superhydrophobic TiO_2_ NP_S_ coated cellulose sponge (superhydrophobic cellulose sponge) was obtained and dried in air for at least 2 h before testing.

### Characterization

The morphological structures of the fabricated cellulose sponge and TiO_2_ NP_S_ coated cellulose sponge were assessed by Scanning Electron Microscopy (SEM, Philips Co., Ltd., Holland). All the samples were cut to 5 mm × 5 mm coupons and coated with a thin layer gold using sputtering for better conductivity before use. The chemical constituents of original and OTMS modified TiO_2_ were analyzed by X-ray photoelectron spectroscopy (XPS, ESCALAB250 spectrometer). Contact angle measurement apparatus (DSA30, Kruss, Germany) was carried out to identify the hydrophobicity and oleophilicity of TiO_2_ NP_S_ coated cellulose sponge. The measured water droplets and oil droplets were all 5 μL, and each sample was measured at least five different positions to obtain the water contact angle (WCA) and oil contact angle (OCA) values.

### Oil-water selectivity

The oil-water selectivity of the superhydrophobic cellulose sponge was examined by using soybean oil (*ρ* < *ρ*_water_) and chloroform (*ρ* > *ρ*_water_) (as representative oil candidates), which were dyed yellow for observation, respectively. Subsequently, a piece of sample was immersed into the above oil/water mixture and then the oil-water selectivity was observed.

### Oil-water separation

The performance of the as-prepared superhydrophobic cellulose sponge was evaluated by the oil-water separation efficiency. The sample disc with a radius of 30 mm and 3 mm thickness was pre-wetted by oil or organic solvent, and then fixed between the two glass tubes. The oil-water mixture was poured onto the superhydrophobic cellulose sponge and the oil-water separation process was driven by its own gravity. Testing oils or organic solvents including Chloroform, Hexane, Kerosene, Toluene and Soybean oil were used and dyed with blue. Meanwhile, the water was dyed with red for easy observation.

### Chemical stability test

The chemical stability of TiO_2_ NP_S_ coated cellulose sponge was examined according to the works of Li and his collaborators^40, 41^, in which the values of water contact angle (WCA) was measured using the water droplets with pH ranging from 1 to 14. In addition, the as-prepared superhydrophobic cellulose sponge was also tested by immersing into the corrosive liquids such as HCl (1 mol/L), NaOH (1 mol/L), salt solution (1 mol/L NaCl) and organic solvent such as chloroform and toluene, for 24 h respectively. After being dried, the WCA values were measured again.

### Robustness test

The aluminium oxide sandpaper of 600 mesh was chosen as an abrasion surface to test the robustness of the as-prepared superhydrophobic cellulose sponge. The sample weighted 100 g was faced down to the sandpaper and moved for 20 cm along the ruler. The above process was defined as 1 cycle of abrasion. After each cycle, water contact angle was measured to evaluate the wettability of the sample.

### Recycling test

After the oil-water separation test finished, the oil contaminated superhydrophobic cellulose sponge could be reused by rinsing with ethanol and water thoroughly. The cleaned superhydrophobic cellulose sponge was dried for 2 h in an oven (60 °C), followed by CA values measurement to test the superhydrophobicity of the sample.

## Electronic supplementary material

Supplementary Information

Movie S1

Movie S2

Movie S3

Movie S4
