# Supplementary material for: A robust superhydrophobic TiO2 NPs coated cellulose sponge for highly efficient oil-water separation
Source: Sci Rep. 2017 Aug 25;7:9428. doi: 10.1038/s41598-017-09912-9 (PMC5572709; doi:10.1038/s41598-017-09912-9)
Supplement: Supplementary file 1 — Supplementary Information [file 41598_2017_9912_MOESM1_ESM.doc]

**A robust** **superhydrophobic TiO_2_ NPs coated cellulose sponge for** **highly efficient oil-water separation**

**Hui Zhang^1^, Yuqi Li^1^, Zexiang Lu^1^, Lihui Chen^1,*^, Liulian Huang^1^ and Mizi Fan^1,2,*^**

^1^College of Materials Engineering, Fujian Agriculture and Forestry University, Fuzhou 350002, China.

^2^Nanocellulose and Biocomposites Research Centre, College of Engineering, Design and Physical Sciences, Brunel University, UB8 3PH, UK.

^*^E-mail: fafuclh@163.com; mizi.fan@brunel.ac.uk

**Supplementary Movie Captions**

**Movie S1.** The as-prepared super-antiwetting surface with low adhesion.

**Movie S2**. Water droplets dripping and sliding on the surface of TiO_2_ NPs coated cellulose sponge.

**Movie S3.** The separation process of chloroform-water mixture (oil dyed with blue and water dyed with red for easy observation).

**Movie S4.** Scratch test (the aluminium oxide sandpaper of 600 mesh used as an abrasion surface).

**Supplementary Figures**


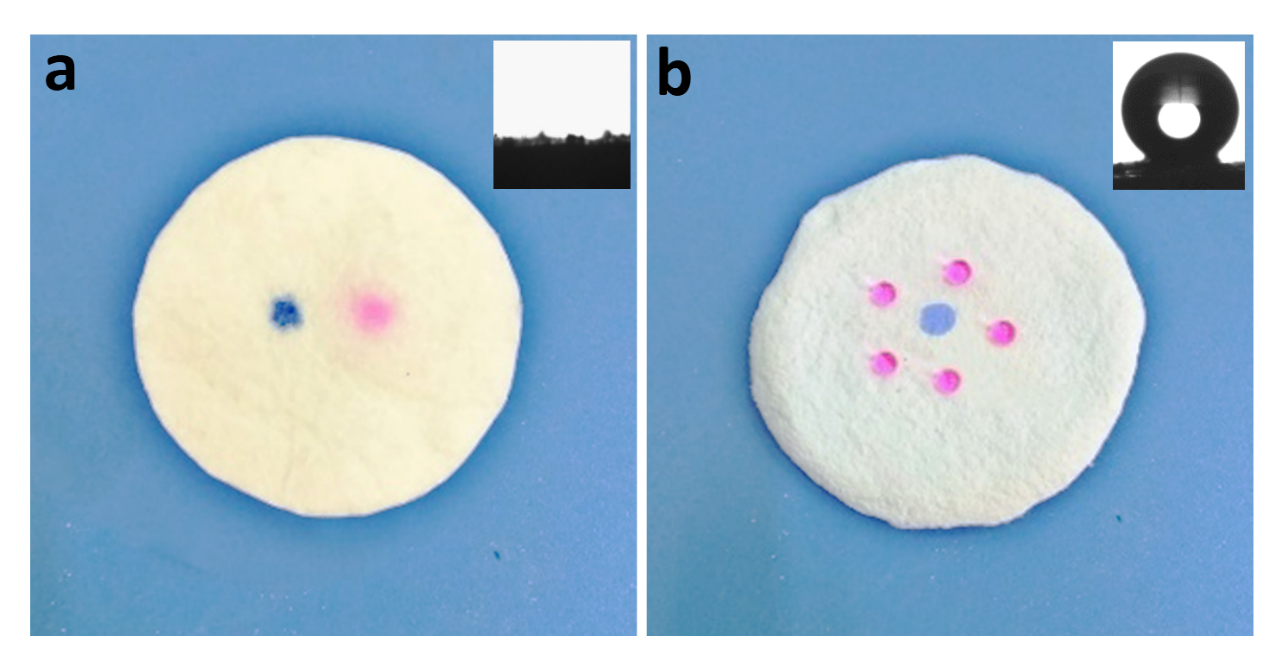


Figure S1. Wetting properties of water droplets and oil droplets on the (a) original, and (b) TiO_2_ NPs sprayed cellulose sponge surface


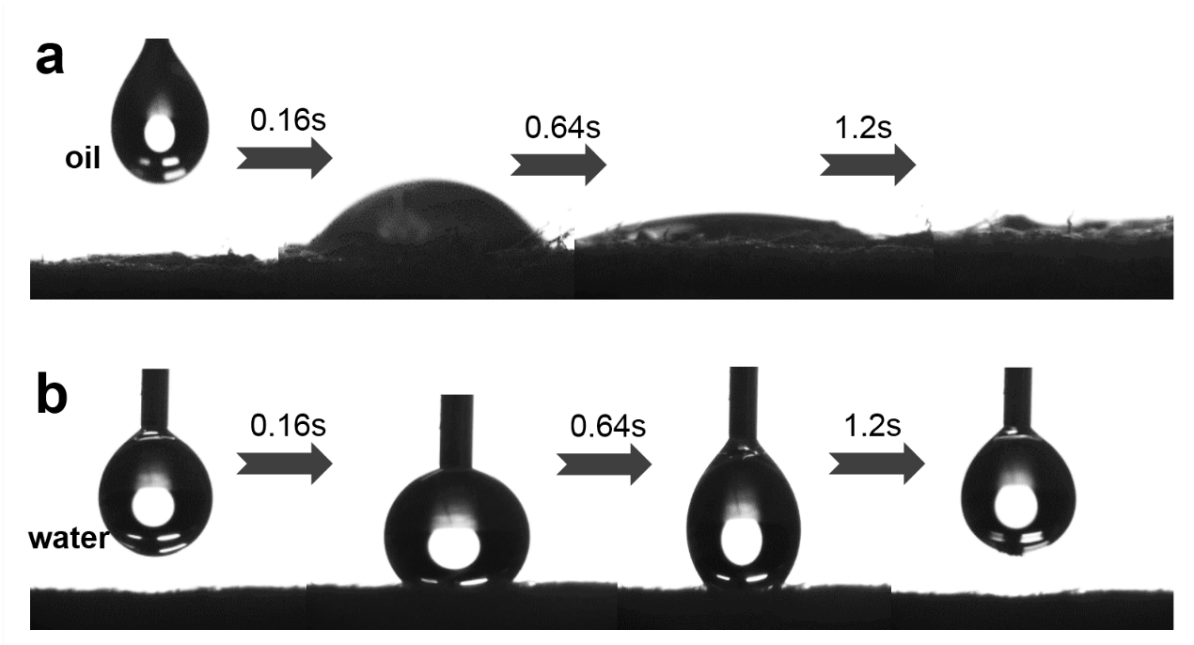


Figure S2. Dynamic wettability of oil (a) and water (b) droplets on the fabricated cellulose sponge


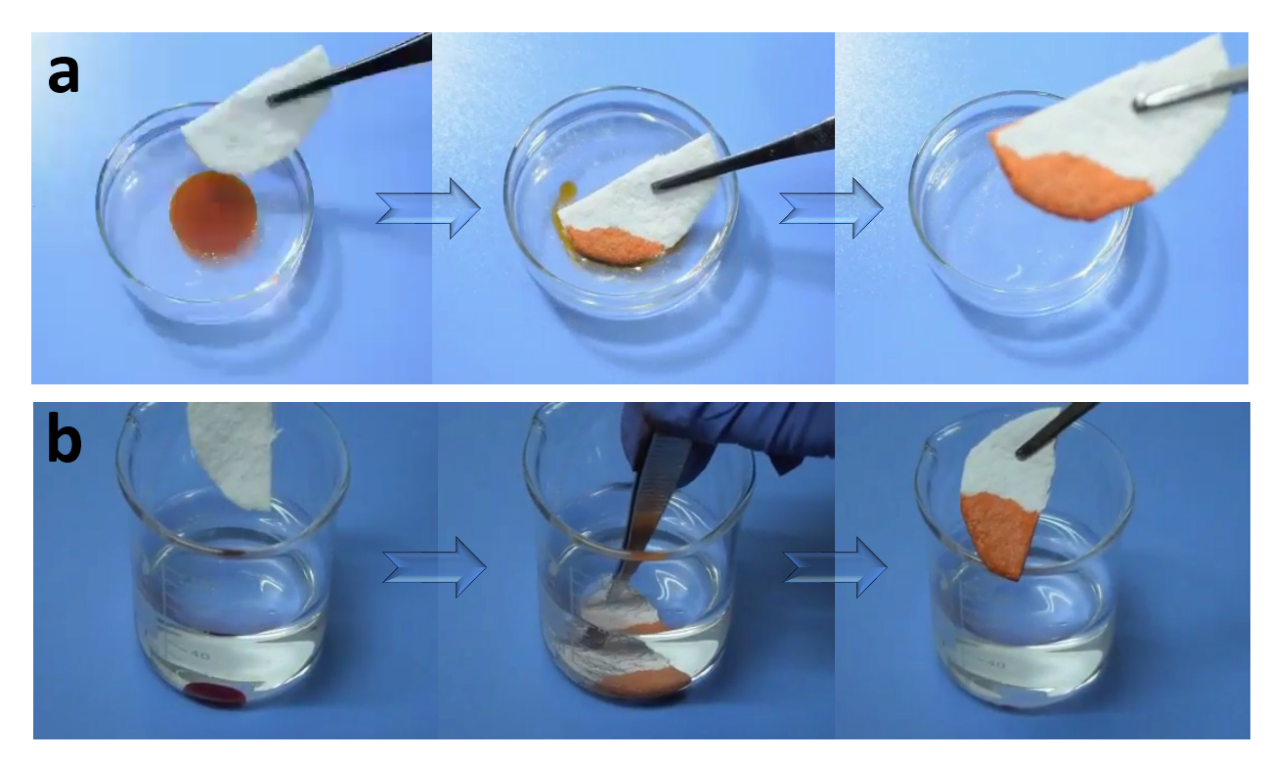


Figure S3. Absorption processes of (a) oil (soybean oil) and (b) organic solvent (chloroform) using the as-prepared cellulose sponge


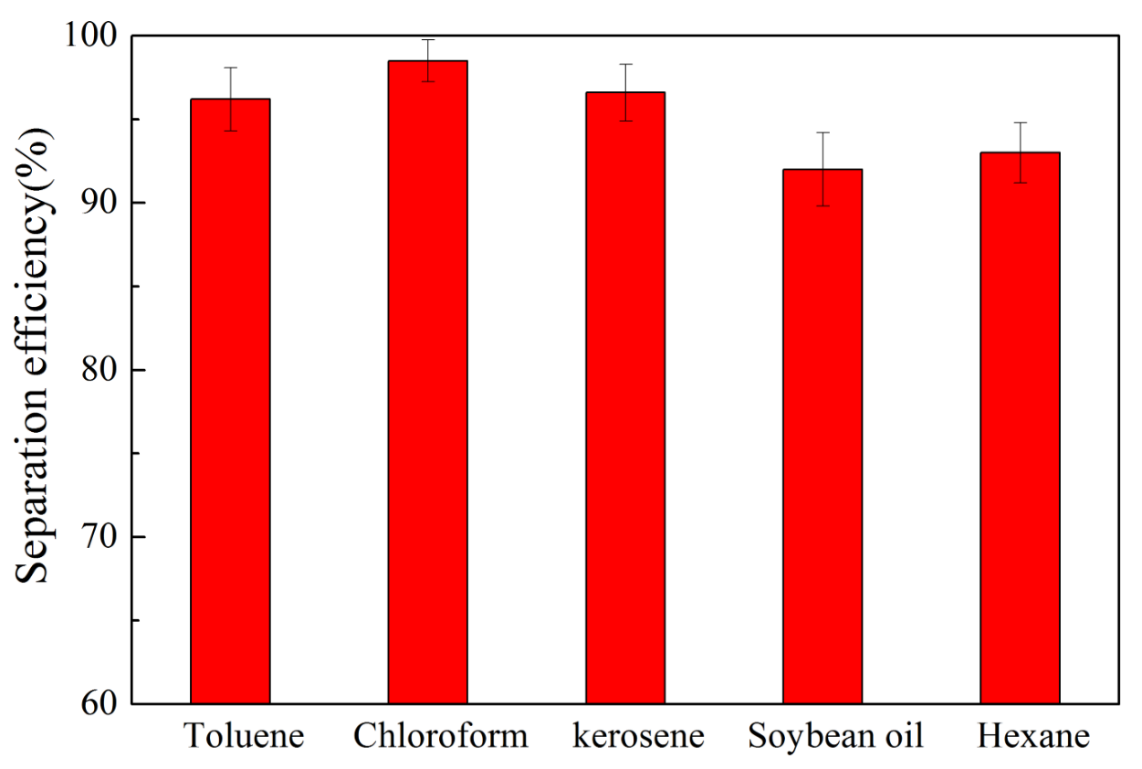


Figure S4. Separation efficiency for various oils-water mixture


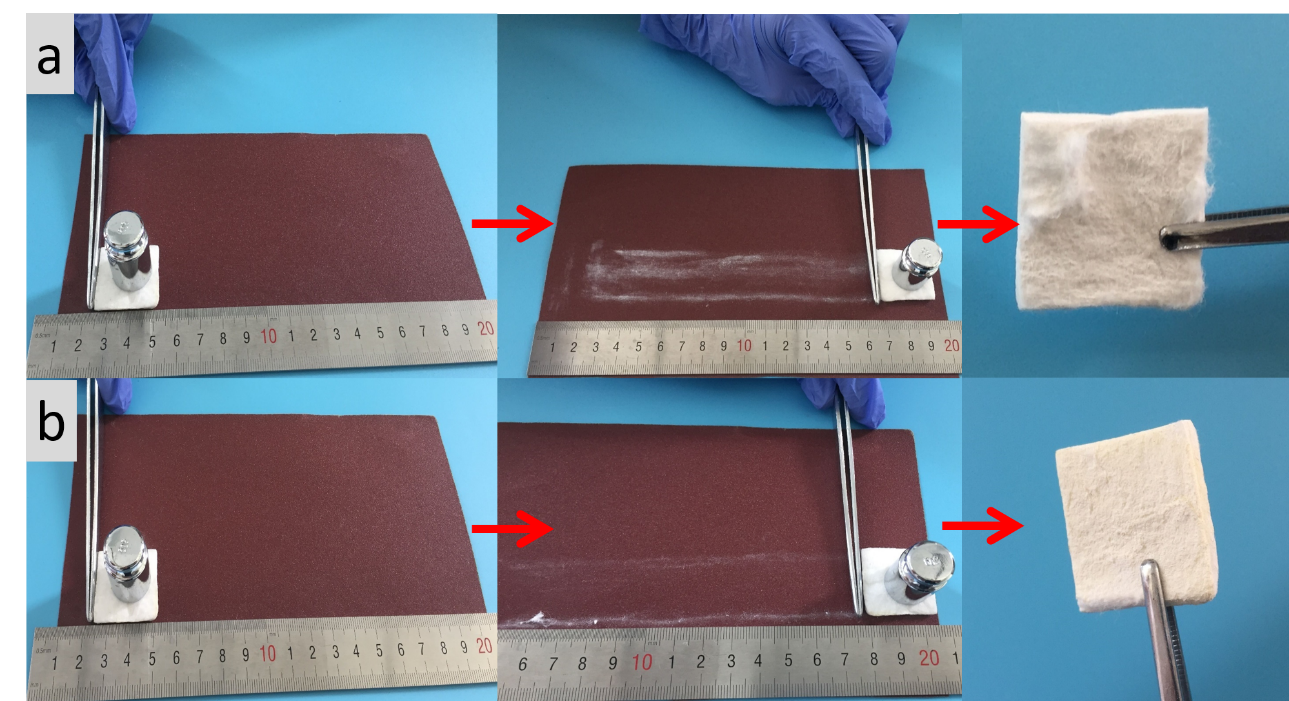


Figure S5. The sandpaper abrasion test (a) TiO_2_ NPs were directly sprayed onto the cellulose sponge surface, (b) TiO_2_ NPs were sprayed onto the adhesive treated cellulose sponge

It can be seen from Fig. S5b, after abrasion with sandpaper, the as-prepared sample treated with adhesive suffered from slight destruction and only a few TiO_2_ NPs fall off from the sample surface. While the sample treated without adhesive suffered from severe destruction and some fragments of the coated fabrics protruded out of the fabric surface (Fig. S5a).


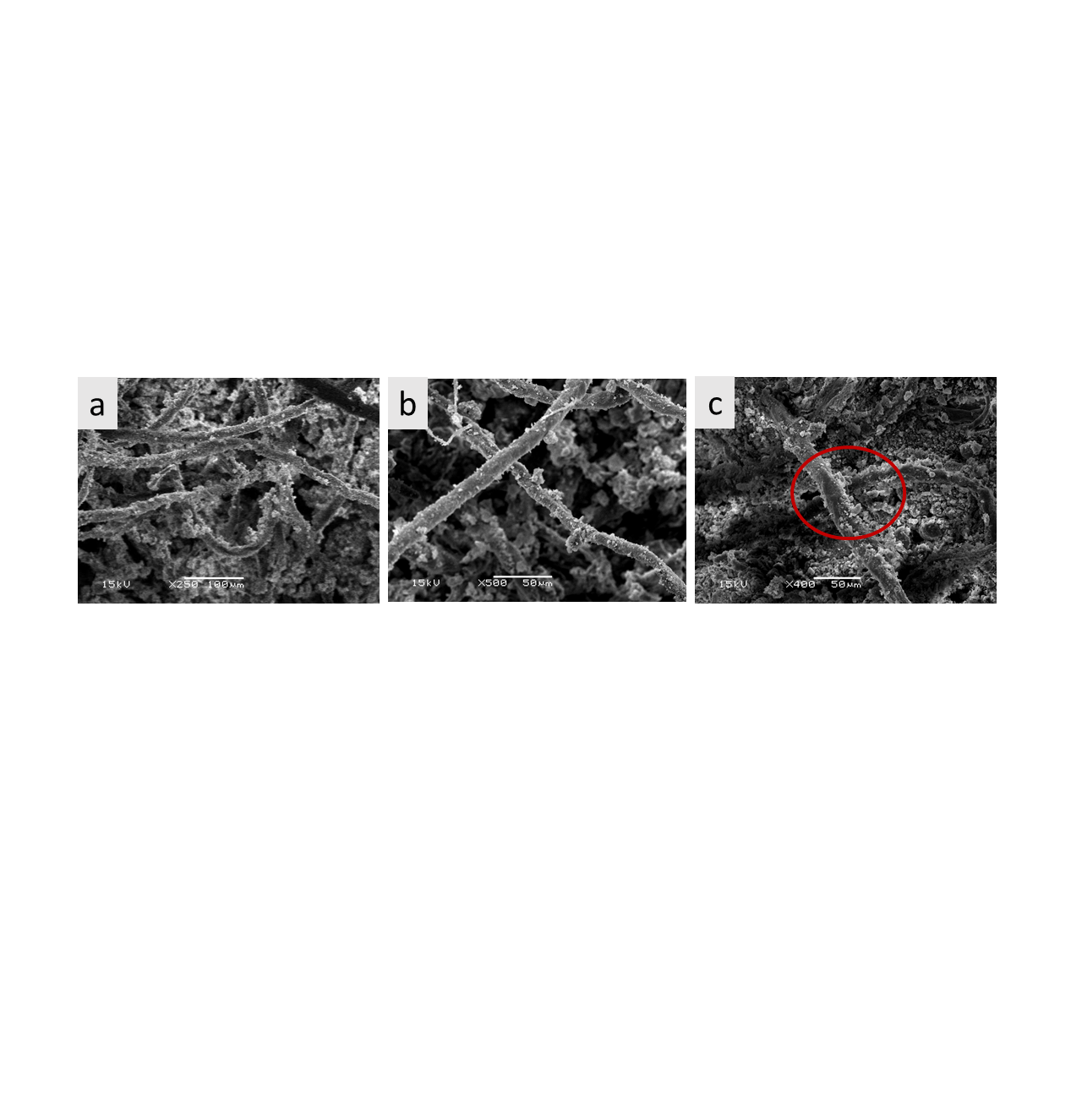


Figure S6. SEM of the as-prepared sample (a, b), and the scratched sample(c)

Fig. S6a shows that the TiO_2_ NPs are randomly distributed and close-packed over all treated fabrics and aggregated around the spaces of interfibers of sponge. As can be seen from Fig. S6b, TiO_2_ NPs were well “protected” by the adhesive and rough structure of cellulose fibers. The surface morphology of the scratched sample in Fig. S6c shows that most of TiO_2_ NPs remain on the surface of the fiber, except for some slight damage on the surface of fibers (in the red region).

According to Fig. S5 and S6, we inferred that the robustness of the as-prepared sample may be ascribed to the adhesive and coarse structure of cellulose sponge, which may make the nanoparticles fixed and embedded into the fibers firmly.


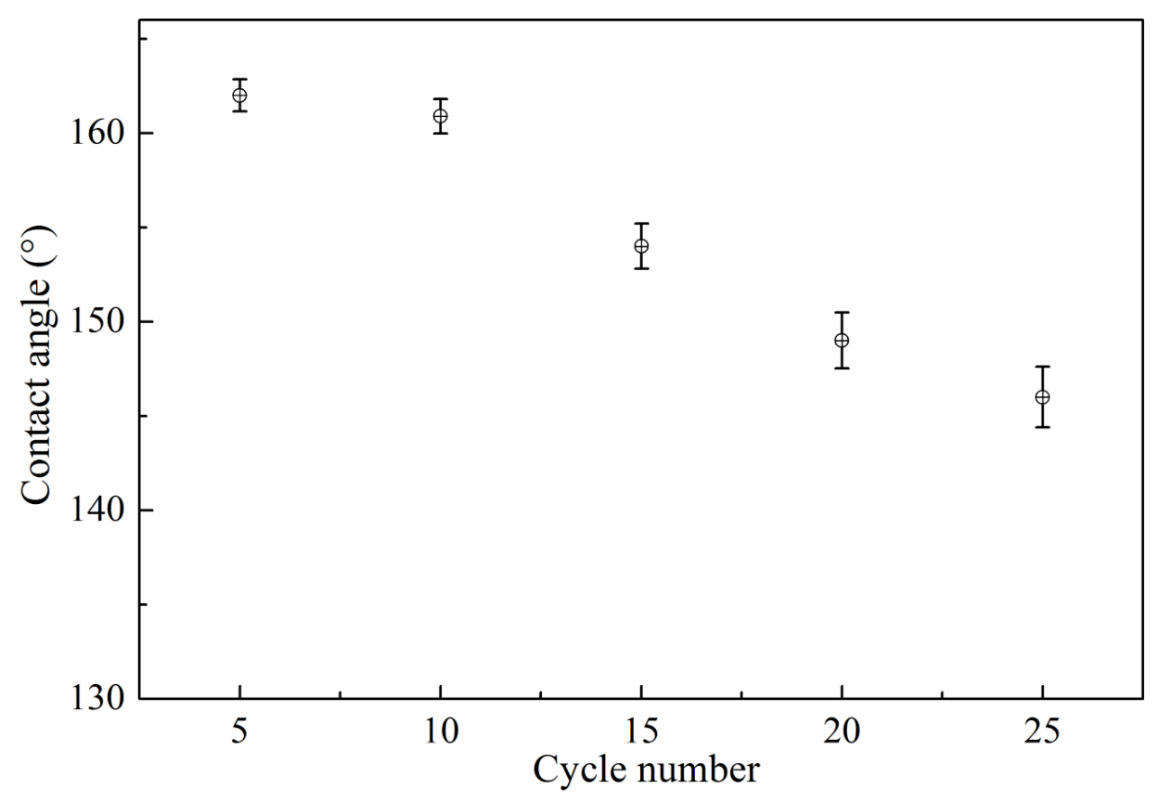


Figure S7. Variation of WCA versus cycle numbers of oil-water separation
